# Adaptive divergence for a drought resistance related trait among invasive Saltcedar (*Tamarix* L.) populations in southwestern US: Inferences from *Q_CT_* - *F_CT_*

**DOI:** 10.3389/fpls.2022.997805

**Published:** 2022-11-14

**Authors:** Soo-Rang Lee

**Affiliations:** Department of Biology Education, College of Education, Chosun University, Gwangju, South Korea

**Keywords:** species invasion, leaf senescence, local adaptation, quantitative traits, *Q_ST_* - *F_ST_*, *Tamarix*

## Abstract

Biological invasion poses several biotic and abiotic challenges due to abrupt distribution shifts. Invasive species may benefit from local adaptation responding to environmental stresses during colonization. Saltcedar (*Tamarix*), a notorious invasive shrub in the western US introduced from Eurasia may have adapted to low rainfall as the species widely occupies the arid land throughout the southwestern US. We investigated variation of quantitative traits in saltcedar between two regions exhibiting opposing average annual precipitations under experimentally manipulated water treatments to test local adaptation. We measured eight quantitative traits, proxies for fitness and genotyped 64 individual samples using genotype by sequencing technique. To test local adaptation, we applied *Q_CT_* - *F_CT_* test based on null distribution of *F_CT_* estimated from 2,697 genome-wide SNPs and *Q_CT_* estimated for the eight phenotypic traits measured. Saltcedar in the southwestern US exhibited a significant interaction between the degree of leaf loss (biomass loss by senesced leaves to total biomass) under simulated drought conditions and the origins from which the genotypes were collected, either relatively high or low rainfall regimes. The divergence found in leaf loss was significantly greater among regions than the expected given the genetic divergence on neutral loci suggesting signature of local adaptation responding to drought. The results demonstrate adaptive potential of saltcedar populations to extreme drought. As extreme aridity is often predicted in climate models across the southwestern US, the western saltcedar genotypes locally adapted to drought may further expand their ranges in this region.

## Introduction

A key to understanding invasion success is to determine what mechanisms enable some colonists to deal with environmental stresses and become successful invaders while many colonists fail to survive in a novel range. Invasive species may cope with the limiting factors in several ways including the following; first, general purpose genotypes (Baker, 1965; van Kleunen et al., 2015) and second, rapid adaptation (Lee, 2002; Maron et al., 2004; Pysek et al., 2009; Colautti and Lau, 2015). Baker (1965) proposed the term, ‘general purpose genotype’ to describe an invasive species with the ability to colonize a wide range of environmental conditions through phenotypic and developmental plasticity. Such species would successfully colonize a new habitat or expand their range without requiring adaptation (Baker, 1965; Parker et al., 2003). In support of this hypothesis, a review of 14 comparative studies between native and invasive species demonstrated that the invasive species are generally more plastic for traits related to fitness than the native species (Richards et al., 2006). Similarly, a more recent meta-analysis compared 75 invasive-native species pairs showed higher phenotypic plasticity in the invasive species than the native species (Davidson et al., 2011). However, as demonstrated in the study whether the increased plasticity is adaptive remains unclear (Davidson et al., 2011). Furthermore, general purpose genotypes may not become successful invaders when the new habitat is full of native competitors or is poor in resources. In a comparative study, over 10 invasive plant species with high physiological plasticity did not outperform the native plants in resource poor conditions (Funk and Vitousek, 2007).

Alternatively, the invasive species may benefit from adaptation to cope with the novel environmental challenges in a new location (Lee, 2002; Blair and Wolfe, 2004; Colautti and Lau, 2015; Corliss and Sultan, 2016). Adaptation is often hypothesized to play important role for successful invasion. There is a growing number of studies investigating the role of local adaptation, particularly during range expansion (Maron et al., 2004; Buswell et al., 2011; Colautti and Barrett, 2013; Nguyen et al., 2016). However, it is challenging to empirically test local adaptation, therefore evidence of adaptation responding to varying environmental gradients is still limited (Colautti and Lau, 2015). In a recent review, Colautti and Lau (2015) demonstrated that divergence between expanding populations has rarely proven to be adaptive despite the comparable quantitative trait divergence found among populations within introduced region. Nevertheless, in one of the few well-studied cases of adaptation during range expansion, Colautti and Barrett (2013) showed that invasive *Lythrum salicaria* L. (Lythraceae) evolved earlier flowering time at its northern invasion front suggesting adaptive evolution of phenology in the northern genotypes. Adaptation may contribute to ecological range expansion of colonists and increase overall invasiveness, but further empirical studies are in need to prove the role of adaptation.

Adaptation to extreme aridity in invasive species is of great concern as global climate models predict severe and widespread aridity in the next 30 to 90 years across many ecosystems (Dai, 2013). Nguyen et al. (2016) investigated five years of drought treatment effect on two invasive plants [*Avena barbata* Pott ex Link (Poaceae) and *Bromus madritensis* L. (Poaceae)] and found regional phenotypic trait divergence responding to the water treatment (Nguyen et al., 2016). However, the study failed to prove if the divergence pattern is the direct result of local adaptation as the similar pattern can be generated by strong influence of random drift associated with historical changes in demography.

Common garden and reciprocal transplanting are the most common approaches to assess genetically based phenotypic variation among populations (Clausen et al., 1940; Linhart and Grant, 1996; Colautti and Lau, 2015). Phenotypic divergence among populations might be caused by either the random chance or responses to selection (Whitlock, 2008). To test local adaptation, *Q_ST_*-*F_ST_* tests can be implemented (Spitze, 1993; Whitlock, 2008). *Q_ST_* is a metric measuring quantitative traits divergence among populations while *F_ST_* is a measure of genetic differentiation among populations (Whitlock, 2008). The test is based on the prediction that if the genetic variance underlying a phenotypic trait is purely additive, genetic divergence among populations on a set of selectively neutral phenotypic traits (*Q_ST_*) is expected to be the same as *F_ST_* for neutral loci (Lande, 1992; Whitlock, 2008). Under this assumption, local adaptation can be inferred when the *Q_ST_* value for a trait is a significant outlier against the genome-wide distribution of *F_ST_*. The interpretation of this test, however, may be biased if the loci used for calculating *F_ST_* are not neutral (Whitlock, 2008). The neutrality issue can partially be addressed by using multiple markers randomly sampled across the whole genome (Leinonen et al., 2013).

The southwestern US has experienced severe and extended drought over the past few decades and aridity in the region is expected to become more extreme based on a climate model (Cook et al., 2015). Saltcedars (*Tamarix* L., Tamaricaceae; here referring to the morphologically indistinguishable hybrid continuum between *Tamarix ramosissima* Ledeb. and *T. chinensis* Lour.) are widespread invasive species in the Western US (Friedman et al., 2005; Gaskin and Kazmer, 2009; McShane et al., 2015). Since saltcedar was first introduced into the US from Eurasian countries in the 1800’s as ornamental shrubs (Bowser, 1958; Robinson, 1965), populations have successfully occupied a variety of temperature and precipitation gradients in the western US. Saltcedar showed not just plasticity but also genetic divergence on root investment in seedlings among regions across a latitudinal gradient from 33°20.5´ N to 46°08.4´N (Sexton et al., 2002). Also, among four plants in the desert southwest, saltcedar was the best competitor, with higher drought tolerance and greater root growth than the other three riparian species (Cleverly et al., 1997). With the traits associated with high drought tolerance, saltcedars might have locally adapted to extreme aridity of desert environment in the southwestern US.

**Figure 1:**
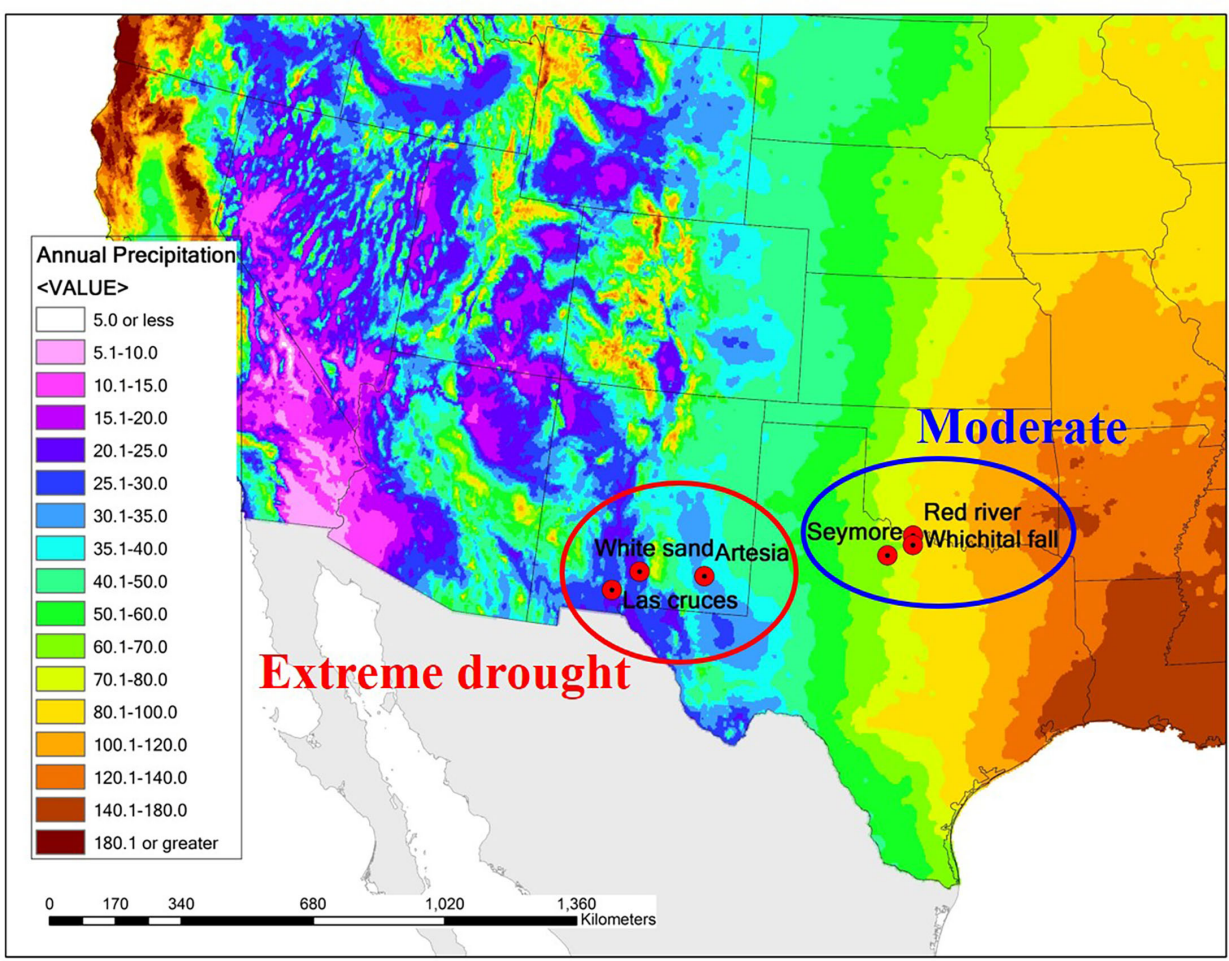
A map of the sampling sites for saltcedar genotypes used in the common garden study. Color gradients on the map represents the average annual precipitation (cm) for the past 30 years based on USDANRCS climate data. Populations sampled in the region with moderate environment with average annual precipitation of ~1000mm are circumscribed in blue, whereas populations sampled in the region with drought environment with average annual precipitation ~200mm in red circle.

In this study, I investigated whether the quantitative trait divergence among saltcedar populations in central Texas and central New Mexico, two regions with large differences in rainfall, were consistent with expectations of local adaptation. The southwestern US exhibits a strong east-to-west rainfall gradient (**Figure 1**), and saltcedar occurs throughout this area. If saltcedars are locally adapted, I expect genotypes from low rainfall environments to exhibit better performance for fitness-related traits than genotypes from high rainfall environments when both are grown in a low soil moisture environment. I also expect the genotypes from high rainfall environments to exhibit better performance for fitness related traits than genotypes from low rainfall environments when both are grown in a high soil moisture environment.

## Materials and methods

### Sample collection and greenhouse experiments

I collected saltcedar samples in two regions differed in average annual precipitation: Central Texas, with an average annual precipitation about 1,000 mm (hereafter called as moderate environment region), and New Mexico with an average annual precipitation about 200 mm (hereafter called as drought environment region; **Figure 1**). Within each region, I collected stem cuttings from 15 genotypes for each of 3 different populations (45 genotypes per region, 90 genotypes total; see **Table 1** for the population information) in mid of March, 2014. The populations from the Central Texas (moderate environment region) were situated in riversides and/or riverbanks, whereas the New Mexico populations (drought environment region) were located in grasslands and a dessert (Whitesand). Given the frequent vegetative growth observed in saltcedars (di Tomaso, 1998), each plant was separated by at least 30m to avoid sampling of genetically identical clones. All collected samples from the populations selected were diploid hybrids (2n=24) between *Tamarix ramosissima* and *T*. *chinensis* (Gaskin and Kazmer, 2009; Lee, 2016). Although these collected genotypes show characteristics of a homoploid hybrid species (Abbott et al., 2003) , in this study I will refer to the operational taxonomic unit (OTU) as saltcedar (Friedman et al., 2011) due to lack of formal diagnosis. Stem cuttings were removed from the newest branch by clipping. Subsequently I cut it into 5 pieces, each about 30 cm in length. Samples were stored at 4°C in zip lock bags for 5 days before propagation.

**Table 1:** Summary statistics of genetic diversity and regional information of sampling sites with geographical position.

Drought condition	Sampling location	Latitude	Longitude	*N*	*He*	*Na*
Moderate (Central Texas)	Witchita Falls	33.84	-98.56	14	0.33 [0.003]	1.95 [0.004]
	Seymour	33.58	-99.27	5	0.27 [0.003]	1.76 [0.008]
	Red River	34.06	-98.57	11	0.31 [0.003]	1.92 [0.005]
Drought (New Mexico)	White Sands	32.80	-106.13	11	0.31 [0.003]	1.94 [0.005]
	Las Cruces	32.33	-106.83	13	0.27 [0.003]	1.92 [0.005]
	Artesia	32.84	-104.32	10	0.27 [0.003]	1.84 [0.007]
Total				64	0.29 [0.001]	1.89 [0.002]

*N* represents the number of genotypes used for moisture stress experiments. *He* and *Na* denote the expected heterozygosity and number of alleles averaged over 2,697 SNP loci.

A greenhouse study with a completely randomized design was performed to test whether the field collected saltcedar genotypes from the two different precipitation environments differed in their response to water stress. Two replicates of each of 90 genotypes were rooted in Ray Leach Cone-tainers (Stuewe and Sons Inc., Oregon, USA) with mixture of coarse peat moss, vermiculite and perlite (1:1:1 ratio) in the Texas Tech Biology greenhouse. After several attempts, 10 of 90 genotypes failed to develop roots from any replicated stem cutting and were eliminated from the experiment. After 4 weeks, I transplanted two replicates from each of the 80 genotypes into 8 ℓ plastic pots (Gro Pro Nursery pot, Sunlight Supply, Inc, Washington, USA) with field collected soil (Texas Tech agricultural farm and autoclaved at 121°C) and cultivated them for 4 weeks prior to experimental manipulation. I sampled the soil from about 30 cm below the surface for each population and used the collected soil for cultivating the genotypes of the same population. I watered the plants daily using tap water and fertilized once per week with commercial water-soluble fertilizer (5-5-15 Peters Excel Cal-mag Grower). Due to mortality during cultivation, total of 64 genotypes were successfully grown and used in the drought experiment (**Table 1**). One replicate of each genotype was randomly assigned to one of two different water regimes: 1) water-stressed, with soil field capacity between 20 to 40%, and 2) well-watered, with soil field capacity between 70 to100%. The pots were watered twice per day until the soil was saturated with water in the well-watered group. For the drought group, volumetric water content of soil was measured every day with soil moisture meter (Hydrosense, Campbell Scientific, Australia). If the soil field capacity fell below the threshold, assigned water calculated was given to the plants based on the water content. Three weeks after the initiation of water treatments and until the final harvest, I collected all leaves that dropped from the plants. These leaves were oven dried (65°C for 24 hours) and weighed for a measure of leaf loss. After eight weeks of growing under the two opposing water treatments (on July, 2014), I harvested the plants and measured additional five traits. In total eight phenotypic traits were included for the final analysis: 1) above ground height, 2) root biomass, 3) shoot biomass, 4) total biomass, 5) biomass ratio, 6) stem diameter, 7) number of stems branches, and 8) leaf loss. The stem diameter measurement was conducted on the thickest part of a main stem using a calliper. These traits were considered as the proxy for fitness given the short duration of the experiment and the long lifespan of *Tamarix* trees. For the biomass measures, I separated above ground shoots and below ground roots and dried them at 65°C for 24 hours before weighing them. Total biomass was calculated as the sum of shoot and root biomass. I included all leaves collected for leaf senescence measurements in the measures of shoot and total biomass. For the biomass allocation, the root to shoot biomass ratio was also calculated.

### Statistical analysis of quantitative traits

Phenotypic traits (height, diameter, number of stems and biomass) that are significantly correlated with the initial size of the plants (P< 0.05; **Table 2**) were analyzed using analysis of covariance (ANCOVA). I used initial size (initial height, initial diameter, initial number of stem) as a covariate in each analysis to account for the influence of correlations. A linear mixed effect model was employed to test whether region of origination, water treatment, or their interaction explained a significant portion of variance in leaf loss (biomass loss by senesced leaves to total biomass), height, number of stems branched from the main stem, shoot biomass, root biomass, stem diameter, and biomass allocation between shoot and root using the Restricted Maximum Likelihood (REML) estimation in R 3.3.0 package ‘lme4’ (Bates et al., 2014; R Core Team, 2021). I calculated the leaf loss using proportion between biomass of senesced leaves and total biomass based on the significant correlation of leaf senescence with the total biomass (r > 0.9; P<0.05). The direct leaf biomass difference before and after the drought experiment could not be calculated because total leaf biomass prior to the drought experiment could not be measured without destroying the plant. Since I was interested in the water treatment, region of origination, and their interaction, I considered the two factors and their interaction as fixed effects and the populations as a random effect nested within each region. Total biomass, shoot biomass, root biomass and diameter were log transformed to meet the assumptions of the linear regression model in linearity of response, identical within-group variance, residual independence, and normality. For the proportions (biomass allocation and leaf loss), I performed arcsine square root transformation in R 4.2.1 (R Core Team, 2021).

**Table 2:** Summary of correlation between initial and final measurements of saltcedar on 5 phenotypic traits. *r* and *P* refer to correlation between the pair of variables and significance of the correlation respectively.

Traits	*r*	*P*
Height - Initial Height	0.439	< 0.001
Diameter - Initial Diameter	0.291	< 0.001
No. of Stems - Initial No. of Stems	0.301	< 0.001
Shoot Biomass - Initial Height	0.200	0.020
Total Biomass - Initial Height	0.193	0.025

### Genotyping and genetic divergence

Genotyping-By–Sequencing (GBS; Elshire et al., 2011) approach was used to collect a genome-wide single nucleotide polymorphisms (SNPs) for 64 genotypes included in the phenotype analysis. For DNA extraction, fresh leaves from greenhouse grown plants were collected and stored at -80°C until the extraction. Genomic DNA was extracted using a DNeasy Plant Mini Kit (Qiagen, Hilden, Germany) and quantified in a Qubit Fluorometer (Thermo Fisher Scientific Inc., Massachusetts, USA). After quality and quantity checks, I submitted DNA samples to the Cornell Institute for Genomic Diversity (IGD) for GBS library preparation and Illumina sequencing (Elshire et al., 2011). IGD prepared the library with the restriction enzyme EcoT221, and sequenced the library using a Hiseq 2000 (Illumina Inc. California, USA).

I called SNPs for 64 genotypes using the UNEAK pipeline in TASSEL 3.0 (Lu et al., 2013). I first trimmed adapters from the raw DNA sequence data (Illumina FASTQ file, 100bp), resulting in 64bp reads which were subsequently aligned to generate clusters of related reads. UNEAK pipeline allowed only 1 base pair mismatch in the cluster membership for quality control. I set the error tolerance rate (ETR) at 0.03, a lower error rate than expected from Illumina sequencing (0.05). Individual genotypes with more than 30% missing calls were removed from the analysis. I used a minimum of 7X read coverage depth for SNP calls to reduce the false discovery rate for heterozygotes (Glaubitz et al., 2014). For the final SNP call, I only included the loci with a minor allele frequency (MAF) of ≥ 0.05 in TASSEL 5.0 to ensure polymorphism (Glaubitz et al., 2014). I further applied a threshold for Hardy-Weinberg equilibrium deviation (*P*< 10e^-6^) to screen out SNPs with extremely high heterozygosity likely resulted from false SNP call or mis-assembly ensuring to keep loci with true structure that exhibit decreased expected heterozygosity.

I estimated genetic diversity parameters, mean expected heterozygosity (*He*) and mean number of alleles (*Na*) over 2,697 SNPs in GENALEX v. 6.5 (Peakall and Smouse, 2012). Analysis of Molecular Variance (AMOVA) was used to estimate how genetic variance was partitioned among regions (*F_CT_*), among populations within regions (*F_SC_*), and among populations overall (*F_ST_*) using Arlequin version 3.5 (Excoffier and Lischer, 2010) . A non-parametric permutation test (1,000 permutations) was used to estimate the significance of variance components. I estimated the distribution of *F_CT_* (genetic differentiation among region) using locus-by-locus AMOVA in Arlequin version 3.5 (Excoffier and Lischer, 2010) .

Hierarchical genetic divergence among regions and among populations within each region were both used to estimate *Q_CT_*. For leaf loss, the only quantitative trait that showed significant divergence among regions in the greenhouse studies, quantitative genetic variance among regions (*Q_CT_*) was estimated as in Keller et al. (2011) with following equations.


$$Q_{CT}=\frac{\sigma_{region}^{2}}{\sigma_{region}^{2}+\sigma_{pop\left(region\right)}^{2}+2(h^{2})\sigma_{resid}^{2}}$$

$\sigma_{region}^{2}$ : The variance among regions,

$\sigma_{pop\left(region\right)}^{2}$ : The variance among populations within regions,

$\sigma_{resid}^{2}$ : The residual variance among individual genotypes

I calculated variance components by fitting water treatment, regions and populations (with each region) as random effects using linear model in R 3.3.0 package ‘varComp’ (Qu et al., 2013; R Core Team, 2021). To ensure independence among molecular markers, SNP markers that are linked (*r^2^* > 0.2) were purged for marker inferred relatedness estimation using Plink 1.9 (Purcell et al., 2007) . I calculated narrow sense heritability (*h^2^*) based on the covariance of phenotype similarity and marker inferred relatedness estimated from a set of 422 unlinked SNPs using Ritland’s MARK ver. 3.1 (available at http://genetics.forestry.ubc.ca/ritland/programs.html). I estimated 95% confidence intervals of *h^2^* by randomly resampling 422 SNP data set with 1,000 bootstrap replicates in R 3.3.0 (R Core Team, 2021) .

To determine whether the regional level divergence on leaf loss is excessive to neutral expectation, I compared *Q_CT_* for the trait against the distribution of *F_CT_* distribution. The significance threshold for *Q_CT_* to be outlier against the *F_CT_* distribution was set to α = 0.05.

## Results

A final data set of 2,697 SNPs representing 64 saltcedar genotypes were retained after the quality control filters. The estimated within-population genetic variation did not significantly differ among 6 populations (*He*=0.27-0.33; *Na*=1.76-1.95 **Table 1**). The genetic variation was the highest in Witchita Falls (moderate environment region; *He*=0.33; *Na*=1.95), whereas the Seymour population (moderate environment region) showed the lowest genetic variation (*He*=0.27; *Na*=1.76; **Table 1**). AMOVA revealed that over 93% of genetic variability was partitioned to within-population genetic variation (*F_ST_* = 0.066; **Table 3**). The genetic variation attributable to differences between the regions was 3.5% (*F_CT_* = 0.036), whereas the variance among populations within each region was 3% (*F_SC_* = 0.032; **Table 3**).

**Table 3:** Partitioning of genetic variance from six saltcedar populations using AMOVA.

Source of variation	Sum of squares	Variance	Fixation index[5%, 95% CI]	Percent of total variance
Among regions (*F_CT_*)	869.068	12.902	0.036 [0.032, 0.039]	3.553
Among populations within regions (*F_SC_*)	1816.023	11.081	0.032 [0.028, 0.035]	3.052
Within populations (*F_ST_*)	19668.096	339.105	0.066 [0.062, 0.070]	93.395

The analysis included populations from 2 regions, 3 populations within each region (see Table 1 for details). All variance components were statistically significant (P< 0.0001).

**Figure 2:**
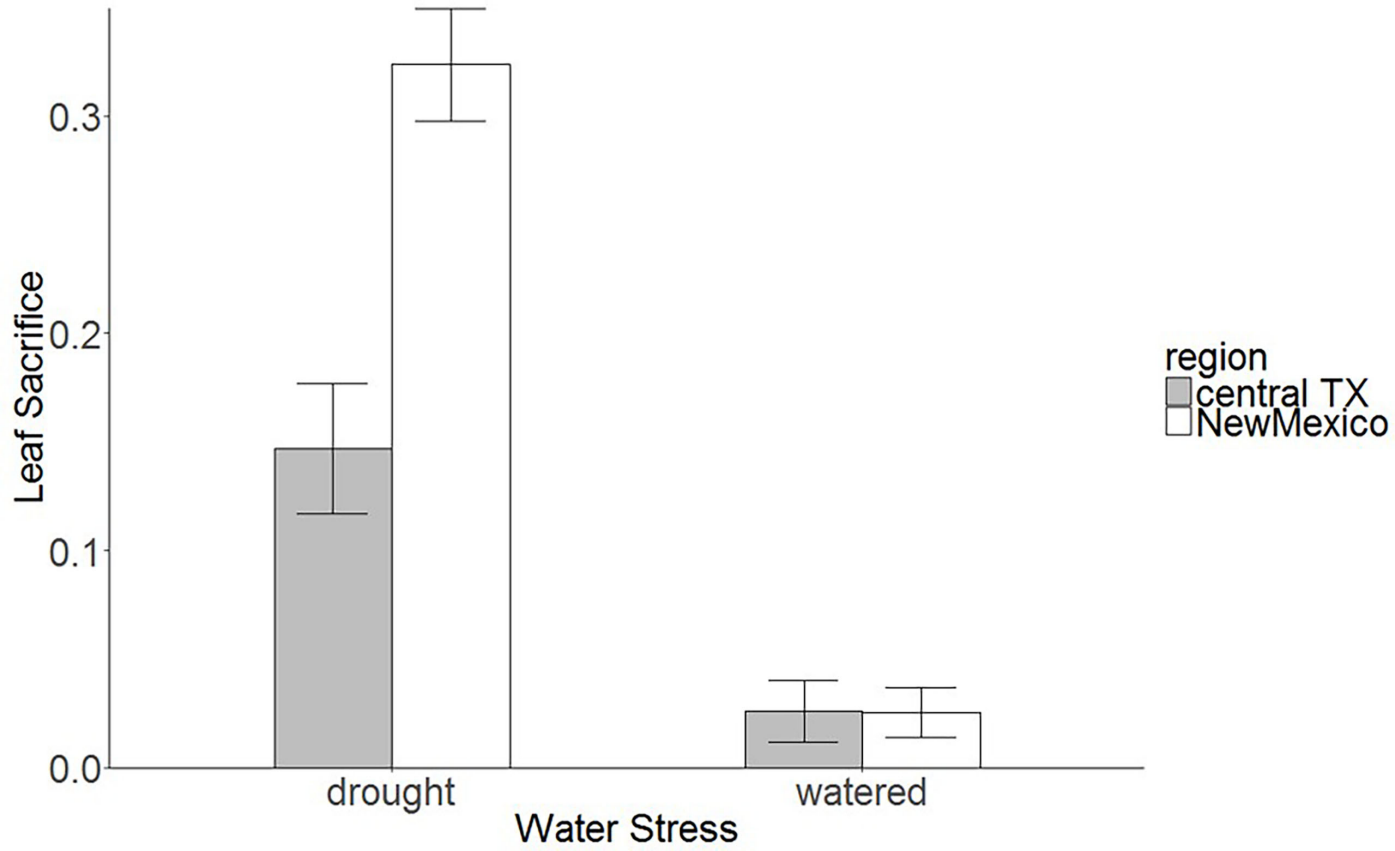
Leaf loss (biomass loss by senesced leaves from total biomass, senesced leaf biomass: total biomass) in different populations of saltcedar from the Central Texas and the desert region of New Mexico. See **Figure 1** for sampling sites. Histograms represent mean proportion of leaves senesced from the total biomass, and error bars represent 95% confidence intervals.

Results from the common garden study showed that the water treatment significantly influenced all phenotypic traits in saltcedar genotypes (**Table 4**). On average, the plant height increased over 80cm in the well-watered group, whereas the water-stressed group increased less than 35cm (estimated from supplementary information 1). Likewise, the stem diameter, the number of stems, the root and shoot biomass showed significantly higher increase in the well-watered group than the water-stressed group (see supplementary information 1). In contrast, most phenotypic traits did not exhibit significant regional differences between the two regions: New Mexico (drought environment region) and central Texas (moderate environment region; **Table 4**). The leaf loss was the only trait that showed a significant interaction between water treatment and region in saltcedar genotypes observed. Genotypes from the Central Texas and New Mexico exhibited the same amount of leaf senescence under the well-watered soil treatment (~ 0.2g and ~ 0.26g respectively), whereas under the drought treatment, genotypes from the New Mexico exhibited 56% greater leaf loss (~ 2g) than ones from the Central Texas (~ 0.8g; **Table 4**; **Figure 2**).

**Table 4:** Summary of Linear Mixed Effects regression model shows differences in drought response between regions with varying soil moisture contents, two contrasting water treatments and their interactions on 8 phenotypic traits in saltcedar.

Traits	*N*	*F*	*P*
Height			
water treatment	125	107.36	< 0.01
Region	2	0.063	0.81
water by region interaction	125	0.144	0.7
Root Biomass			
water treatment	130	36.2	< 0.01
Region	2	0	0.99
water by region interaction	130	0.613	0.44
Shoot Biomass			
water treatment	126	270.36	< 0.01
Region	2	0.21	0.68
water by region interaction	126	0.11	0.74
Total Biomass			
water treatment	126	240.44	< 0.01
Region	2	0.18	0.7
water by region interaction	126	0.18	0.67
Biomass ratio			
water treatment	130	19.53	< 0.01
Region	2	0.11	0.74
water by region interaction	130	0.99	0.32
Stem Diameter			
water treatment	122	5.02	0.03
Region	2	0.63	0.48
water by region interaction	122	1.73	0.19
No. of branches			
water treatment	122	85.3	< 0.01
Region	2	0.01	0.93
water by region interaction	122	0.02	0.89
Leaf loss			
water treatment	127	102.5	< 0.01
Region	2	3.93	0.14
water by region interaction	130	18.31	< 0.01

**Figure 3:**
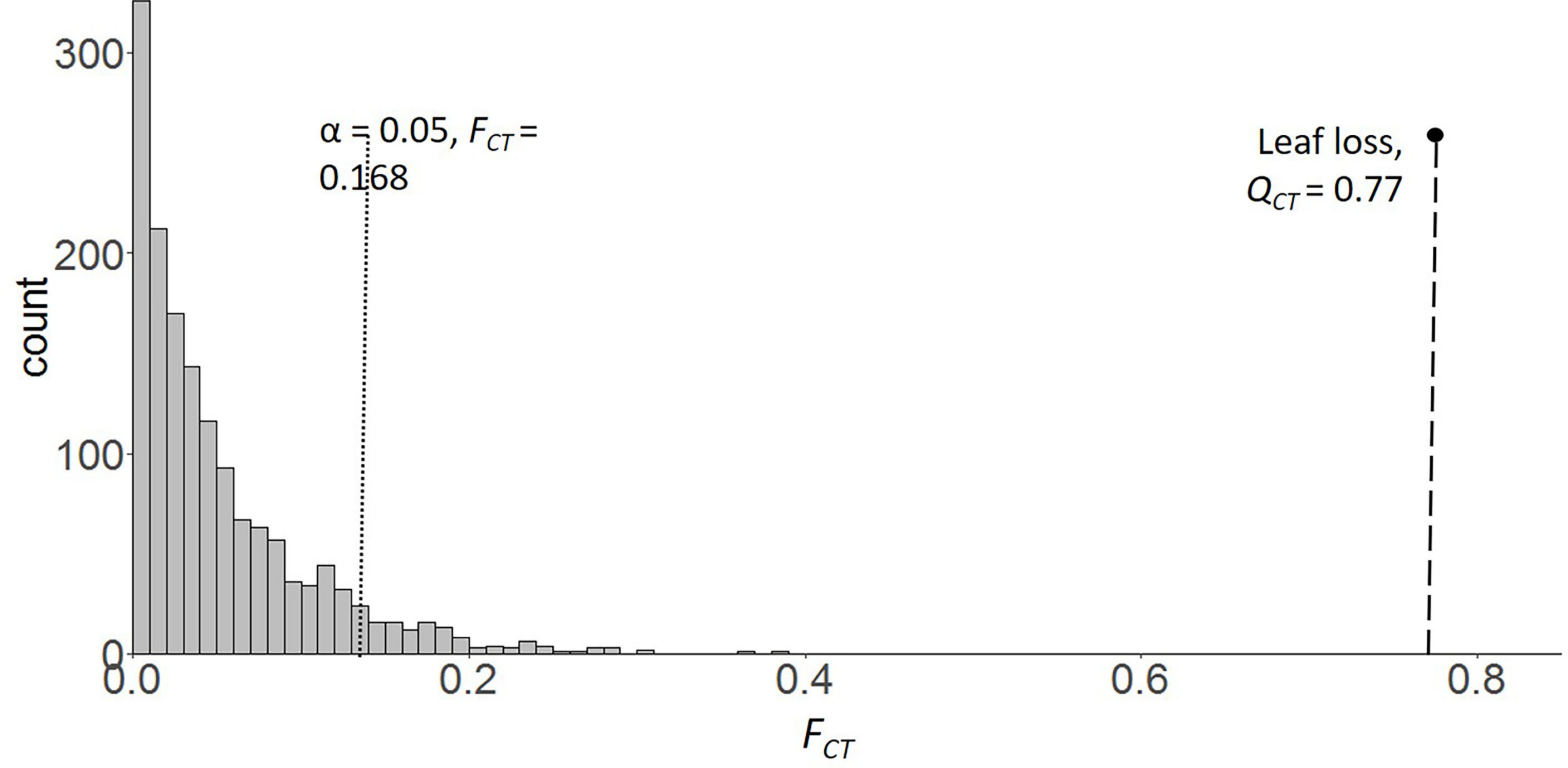
F_CT_ distribution of 2,697 SNP loci from 64 individuals sampled across 6 populations of saltcedar. The vertical dotted line and dashed indicates the significance threshold, α = 0.05 for the F_CT_ distribution and the estimated Q_CT_ of leaf loss respectively.

Heritability of leaf loss was significantly different from zero (*h^2^* = 0.25, 95% CI [0.07,0.43]). Except for *h^2^* of leaf loss, the *h^2^* of the remaining 7 phenotypic traits were low and did not significantly differ from zero (*h^2 =^* 0.014-0.087, all CI values overlap with 0). The estimate of region-level quantitative trait divergence on leaf loss (*Q_CT_*) was 0.77. *Q_CT_* of leaf loss fell far above 95% of the estimated regional variance for neutral genetic markers (*F_CT_*; **Figure 3**).

## Discussion

Local adaptation during species invasion is one of the most important mechanisms for successful colonization and subsequent range expansion (Lee, 2002; Maron et al., 2004; Colautti and Barrett, 2013; Colautti and Lau, 2015). However, detecting selection responding to environmental gradients on a long-lived tree poses a suit of challenges e.g. maternal effects, evolutionary lag times, difficulties of growing under a common garden setting (Wright, 2007; de Kort et al., 2013; Mayol et al., 2020). In the study, I found a significant variation between the two opposing water treatments on all phenotypic traits measured (**Table 3**), which likely is an indication of significant soil moisture effect on the saltcedar’s fitness. The significant quantitative trait divergence together with the inflated *Q_CT_* - *F_CT_* on leaf loss suggested a possibility of selection responding to the precipitation gradients in the American West. Although the small sample size, lack of replications and the probable maternal environmental effects might undermine the result to some extent, the results provided an empirical evidence of the local adaptation playing as a significant contributor to the successful invasion of saltcedar in the arid American West.

Water availability decreases from east to west in the southern US (**Figure 1**). In arid regions such as the Chihuahuan Desert of southern New Mexico and West Texas, soil moisture is a dominant factor limiting vegetation growth and survival. Saltcedars showed higher physiological tolerance to water deficit environments over native competitors, such as *Populus* spp. and *Salix* spp., by deep and fast root development and high cavitation resistance (Cleverly et al., 1997; Pockman and Sperry, 2000; Horton and Clark, 2001; Hultine and Dudley, 2013). With these competitive drought resistant characteristics, saltcedars may plastically adjust to periodic drought. However, if drought is procrastinated as in the Chihuahuan Desert, one of collection sites for drought region, saltcedars may have to locally adapt to the extreme aridity. My study found a regional divergence pattern consistent with local adaptation to extreme drought on leaf loss (**Table 3**; **Figure 3**).

Leaf senescence is a complex and genetically regulated process which contributes to fitness of the whole plant thereby an evolutionarily relevant developmental process (Lim et al., 2007). Notably, saltcedar genotypes collected from extremely arid New Mexico shed more leaves (58%) than the genotypes from wetter soil environment (Central Texas) under 8 consecutive weeks of drought (field capacity 20% to 40%). The trait divergence (*Q_CT_*) among the two extreme aridity conditions was significantly greater than the estimated null divergence (*F_CT_*, **Figure 3**). This suggests that leaf loss (leaf senescence adjusted for total biomass) found in saltcedar may be adaptive to drought, therefore may have contributed to the wide distribution of saltcedar in the arid American West. By dropping more leaves, the New Mexican (drought environment region) genotypes of saltcedar probably would conserve more water and increase cavitation resistance for the stems, a critical organ for plant survival, during the driest season. The increased leaf senescence may also contribute to recycling nutrients for future leaves or flowers.

I inferred adaptive evolution by comparing divergence of additive genetic variation in quantitative traits (*Q_ST_*) to divergence of random samples from molecular markers (2,697 SNPs). To avoid biases of using point estimates i.e. mean of *F_ST_* from a few loci, I generated an empirical *F_ST_* distribution based on large number of SNPs collected across the whole genome (Whitlock, 2008). Although it is probable that a few SNPs are under direct or indirect influence of selection, the *F_ST_* distribution estimated from large SNP data set is representative. Additionally, the approach allows more robust null expectations than the parametric approach for hypothesis testing (Whitlock, 2008; Whitlock and Lotterhos, 2015).

I indirectly estimated the additive genetic variation using quantitative trait variance measured under controlled environment in the greenhouse and marker-inferred narrow-sense heritability (Ritland, 2000). Due to possible increase in residual variance driven by environmental influences even under controlled greenhouse setting, additive genetic variance might be over-estimated. Therefore, estimated *Q_CT_* may be conservative (Whitlock, 2008). Nevertheless, the *Q_ST_* estimate was far beyond the right tail of *F_ST_* distribution rejecting the null hypothesis of neutrality suggesting a signature of local adaptation. However, the divergence may not be the result of adaptive responses to the environmental gradients but the result of maternal effects as there was no sufficient time or generations of the cuttings raised. Unfortunately, controlling the maternal effects by raising over two generations from seeds is not the practical solution for a long-lived tree like saltcedar (Wright, 2007; de Kort et al., 2013). Yet, in this particular phenotype data set, the environmental maternal effects may not be the likely bias for the significant divergence observed. The maternal effects tend to target early life traits e.g. seed mass, germination rate and early growth, which is not the phenotype with the adaptive divergence in this study (de Kort et al., 2013).

## Conclusions

Saltcedars have spread widely throughout the American West since naturalization in the early 1900s (Robinson, 1965; Friedman et al., 2005). My study suggested that rapid adaptation might have largely contributed to saltcedar survival for moisture scarcity, a dominant environmental stress in the southwestern US. As climate change progresses in the American West, saltcedar genotypes with greater adaptive potential in the arid region may further expand their range replacing native riparian vegetation.

## Data availability statement

The datasets presented in this study can be found in online repositories. Phenotypic data have been accessioned at Dryad (datadryad.org) under the title: "Quantitative trait divergence and local adaptation to drought in the invasive Saltcedar (Tamarix)". Phenotypic data are provided as **Supplementary Material Table**.

## Author contributions

The author confirms being the sole contributor of this work and has approved it for publication.

## Funding

This study was supported by research fund from Chosun University, 2022.

## Acknowledgments

I am grateful to Texas Tech Biology Department and Texas Tech University Association of Biologists for providing travel funds and greenhouse equipment. Greenhouse manager Jenifer Smith and staff William Barnes have helped with experiment settings and given advices for cultivating. Dr. Yeong-Seok Jo gave great help with collecting samples and greenhouse cultivation. Finally, I give my special thanks to Dr. Olson at Texas Tech, Dr. Jonathan Friedman from USGS for invaluable comments and for the help of revising the manuscript.

## Conflict of interest

The author declares that the research was conducted in the absence of any commercial or financial relationships that could be construed as a potential conflict of interest.

## Publisher’s note

All claims expressed in this article are solely those of the authors and do not necessarily represent those of their affiliated organizations, or those of the publisher, the editors and the reviewers. Any product that may be evaluated in this article, or claim that may be made by its manufacturer, is not guaranteed or endorsed by the publisher.
